# Effect of glide path instruments in cyclic fatigue resistance of reciprocating instruments after three uses

**DOI:** 10.1590/0103-6440202305226

**Published:** 2023-05-15

**Authors:** André Schroder Scherer, Carlos Alexandre Souza Bier, José Roberto Vanni

**Affiliations:** 1 Graduate program in Dental Science, Federal University of Santa Maria, Santa Maria, Rio Grande do Sul, Brazil.; 2 Department of Stomatology, Federal University of Santa Maria, Santa Maria, Rio Grande do Sul, Brazil.; 3 Graduate program in Dental Science, Dental Specialty Center Meridional, Passo Fundo, Rio Grande do Sul, Brazil.

**Keywords:** Endodontics, root canal preparation, fractures

## Abstract

The present study aims to evaluate the effect of different glide path instruments on the cyclic fatigue resistance of reciprocating endodontic instruments after three uses in mandibular molars. Eighteen Wave One Gold Primary reciprocating instruments were selected and randomly divided into three groups according to the glide path instrument: G1 - manual file K #15, G2 - Wave One Glider reciprocating instrument, and G3 (control group) - glide path was not performed. The reciprocating instruments were tested on mandibular molars and subdivided into three other groups: a new instrument, an instrument with a previous single-use, and an instrument with two previous uses. After the endodontic instrumentation, the instruments were subjected to the cyclic fatigue resistance test using an appropriate tool. The data were submitted to the Shapiro-Wilk test, and subsequently the Kruskal-Wallis test with a significance level of 5%. The results showed no statistical difference between the groups. Thus, it was concluded that the creation of a glide path did not affect the cyclic fatigue resistance of the reciprocating instrument. In addition, the reuse of final preparation instruments up to two times proved to be safe since no fractures were observed in the tested instruments.

## Resumo

O objetivo do presente estudo foi avaliar a relação de diferentes instrumentos de glide path na resistência à fadiga cíclica de instrumentos endodônticos reciprocantes de preparo final após três utilizações em molares inferiores. Foram selecionados 18 instrumentos reciprocantes Wave One Gold Primary e divididos de maneira randomizada em três grupos: G1 - glide path utilizado foi uma lima manual K #15, G2 - glide path utilizado foi um instrumento reciprocante Wave One Glider, e G3 (controle) - sem utilização de glide path. Os instrumentos foram testados em molares inferiores e subdivididos em outros três grupos: instrumento novo, instrumento com um uso prévio e instrumento com dois usos prévios. Após os testes, os instrumentos foram submetidos ao teste de resistência à fadiga cíclica por meio de uma ferramenta própria para esse uso. Os dados encontrados foram submetidos ao teste de Shapiro-Wilk para avaliar a normalidade na distribuição da amostra, e posteriormente ao teste de Kruskal-Wallis com nível de significância de 5%. Os resultados demonstraram não haver diferença estatística entre os grupos testados. Com isso, concluiu-se que a criação de um glide path não possuiu efeito na resistência à fadiga cíclica do instrumento reciprocante de preparo final. O instrumento reciprocante de preparo final foi utilizado, e posteriormente reutilizado de maneira segura em três dentes posteriores, visto a não ocorrência de fratura em nenhum dos grupos testados.

## Introduction

The advent and popularization of mechanized systems used for instrumentation of the root canal system brought a number of advantages in daily clinical practice, such as predictability and agility in treatment, reduction of deviations, and maintenance of the original trajectory of the canal ^1^. However, some disadvantages have also stemmed from the use of these instruments, where the main occurrence of accidents is the fracture of nickel-titanium (NiTi) instruments. In an epidemiological study, fracture of these instruments was significantly the most common complication, accounting for more than half of the complications during the treatment ^2^.

The factors that lead a NiTi instrument to fracture are varied, where we can mention the rotation speed, the angle of curvature of the canal, the force used by the operator during treatment, and the number of times that the instrument is used ^3^.

Reuse instruments are one of the factors related to the occurrence of fracture because it generates microstructural, microchemical, and mechanical changes that can be detected in NiTi instruments ^4^. Moreover, every time an instrument is reused it goes through a sterilization process, which is capable of generating changes in the resistance to torsional fatigue, even if this result has no direct correlation with the clinical significance of these data ^5^. Because of this, and for abetter-established safety, manufacturers continue to indicate in the instruments' package inserts that they should be used once only, and immediately discarded after use. However, even with the manufacturers' indications, epidemiological studies show that operators routinely reuse NiTi instruments. Such methods are mainly due to the social, economic, and financial conditions in which these professionals are inserted ^6^ ^,^ ^7^.

On the other hand, technological advancement has been increasing, and the technology implemented in the manufacture of NiTi instruments has been quite significant. The heat treatment of the instruments can be evaluated as one of the main recent advances because this new technology improves the mechanical properties and allows a shape memory of these instruments. Clinically this indicates greater flexibility, the ability to maintain the canal trajectory without causing deviations, and better cutting efficiency ^8^. In the evaluation of cyclic fatigue resistance of reciprocating instruments with and without heat treatment, the results indicate that the instrument with the presence of the metal alloy heat treatment was able to generate better resistance to cyclic fatigue than instruments without this technology ^9^.

Associated to the metallic alloy heat treatment technology, another important factor of technological advance in endodontics was the reciprocating kinematics, which consists in an oscillatory movement in which the instrument rotates a certain degree of rotation in one direction, followed by another certain degree of rotation in an opposite direction until it finishes a complete rotatory movement. As an example of reciprocating movement we can mention the Wave One Gold instruments (Dentsply Mailefer, Ballaigues, Switzerland), which rotate 60º clockwise and 120º counterclockwise until the complete rotation is completed ^10^. When the technologies are associated, the results of resistance to cyclic fatigue of instruments with reciprocating kinematics and alloy heat treatment are superior to instruments that do not have both technologies associated ^11^.

Along with the advancement in preparation instruments, other ways of maintaining safer preparation have been introduced by manufacturers in order to allow safer clinical care. One of these is the glide path instrument, a class of instruments that has been used prior to final preparation in order to generate a free working path so that subsequent instruments can act with their free end. This action is called the glide path and allows the reduction of debris extrusion and the maintenance of the original path of the root canal ^12^.

Of the instruments used for this purpose, the manual Kerr stainless steel files are the most widespread and widely used. However, with the advance in the production of materials destined for specific stages of endodontic treatment, some instruments have been produced for this purpose. These include the Path File (Dentsply Mailefer), a 16.02 rotary kinematics instrument, and also the Wave One Gold Glider (Dentsply Mailefer), with reciprocating kinematics, diameter 15, and variable taper. Both are produced with NiTi alloys and have the advantage over stainless steel hand instruments, the heat treatment process in their manufacture, which increases resistance to cyclic fatigue and also flexibility ^13^.

Glide path instruments with reciprocating kinematics have recently been introduced in daily clinical practice, but data already indicate that these instruments have a higher resistance to cyclic fatigue ^14^, and a good condition to reach the working length with a low rate of deformation and fracture ^15^.

Therefore, due to the technological advancement, which includes glide path instruments, and the significant advance in the manufacturing of instruments, such as reciprocating kinematics and heat treatment of alloys, and mainly due to the social, economic, and financial conditions in which the dental surgeon is inserted, this study aims to evaluate the impact of reusing heat-treated reciprocating endodontic instruments on their cyclic fatigue resistance, associated with the use of different glide path instruments. The clinical relevance of the study is associated with the occurrence of fractures of NiTi instruments during treatment, and also in making guides and protocols for safe reuse.

The null hypothesis points to a non-correlation between the glide path instrument used and the cyclic fatigue resistance of the preparation instrument, as well as the non-occurrence of fractures.

## Material and methods

The study was submitted and approved by the Research Ethics Committee of the Federal University of Santa Maria and is registered under number 45106621.2.0000.5346.

### Sample Size

The sample size calculation was performed considering the relationship between the use of glide path instruments and the cyclic fatigue resistance of the final preparation instrument from a previous study ^16^. The parameters for sample size calculation were based on an effect of 150 cycles for fracture occurrence based on the cyclic fatigue strength of heat-treated reciprocating instruments. Using a standard deviation of 79.10 as a parameter (based on a previous study [16], the group using a glide path instrument and Wave One for final preparation), a significance level of 5%, and a statistical power of 80%, 6 instruments were estimated per group.

Eighteen reciprocating instruments Wave One Gold Primary 25.07 (Dentsply Mailefer) were selected for preparation. The instrument was selected because is currently used for shaping the molar’s root canals, furthermore, it is an instrument with reciprocating motion and presented heat-treated nickel titanium. These characteristics bring this instrument good popularity, in addition to the advanced technology in its manufacture.

**Figure 1:**
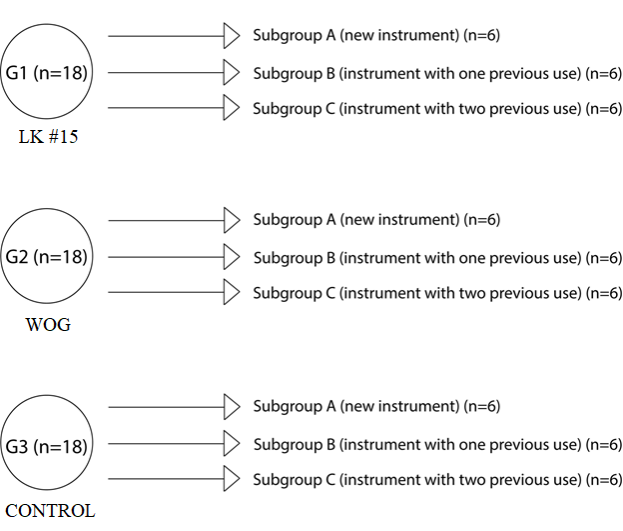
Quantitative and qualitative division of the groups into their respective subgroups.

The instruments were randomly divided into three groups as shown in Figure 1.

### Teeth Selection

Fifty-four mandibular molars with two independent roots, complete apical development, and without previous endodontic treatment, available through the human permanent tooth bank of the Federal University of Santa Maria, were selected. The selection of teeth that participated in the study was based primarily on a radiographic analysis of the specimens. Digital radiographs (RVG5100, Carestream dental, Atlanta, USA) were taken in a buccolingual direction and the curvatures and radius of curvature of the mesial root were measured ^17^. The inclusion criteria were mesial root with mean curvature between 10-20º and radius of curvature between 5.5-16.5mm, and distal root with mean curvature between 0-10º. There were two independent canals in the mesial root with the presence of a single foramen (Vertucci classification type II), whereas the distal root had a single canal. The teeth did not present root caries, external or internal resorption, root fractures, or previous endodontic treatment. After selection, the teeth were randomly divided into the three groups already determined.

### Subgroup Division

In each group, the teeth were randomly subdivided into three other subgroups. Subgroup A was instrumented using a new instrument, immediately after being removed from the blister, with the silicone ring intact. After use, the instruments were washed in running water, packaged, and sterilized by autoclaving (Vitale Class CD 12 liter, Cristófoli, Campo Mourão, Brazil) at 134ºC for 24 minutes ^18^. In subgroup B, the instruments previously used in subgroup A (one previous use) were used. After use, the instruments were washed, packaged, and sterilized as previously described ^18^. Finally, in subgroup C, the instruments previously used in subgroups A and B (two previous uses) were used (Figure 1).

### Chemicomechanical Preparation

A single operator performed the chemicomechanical preparation. Initially, the coronal access of the dental elements was performed using a high-speed turbine and 1014HL diamond burrs (KG Sorensen, Cotia, SP, Brazil). After the coronary access and localization of the root canals, exploration was performed using a K #10 (Dentsply Mailefer) file in the mesiobuccal, mesiolingual, and distal canals until the tip of the instrument was seen in the apical foramen. The instrument was removed and the measurement found was considered the real length of the tooth, and, reducing this measurement by one millimeter, the working length was determined. Group I underwent preparation with a K #15 (Dentsply Mailefer) file in the root canals with a catheterization movement and slight apical pressure until it reached the real length of the tooth. After this exploration, filing movements were performed to make the glide path. Group II underwent glide path preparation with a Wave One Gold Glider (Dentsply Mailefer) reciprocating instrument. The process of use was carried out according to the manufacturer's instructions using the VDW Silver endodontic engine (VDW Dental, Munich, Germany) in the wave one all system in a slight back-and-forth movement, with an amplitude of about three mm until the instrument reaches the predeterminited real length of the tooth. Group III, also called the control group, and underwent only one exploration with a #10 K file (Dentsply Mailefer) up to the real length of the tooth with catheterization movements and light apical pressure. The final preparation instrument for all groups was performed with Wave One Gold Primary 25.07 (Dentsply Mailefer) in the reciprocating motion, using VDW Silver endodontic engine (VDW Dental) in the wave one all system in a slight back and forth movement with an amplitude of about three mm. The operative protocol was repeated until the instrument reaches the working length. The irrigating solution used was 2.5% sodium hypochlorite. The irrigation protocol consisteds of irrigation with back and forth movements, where the needle was inserted as apical as possible, without blocking the walls of the root canal. A volume of 5ml was used at each operative step.

### Cyclic Fatigue Test

**Figure 2:**
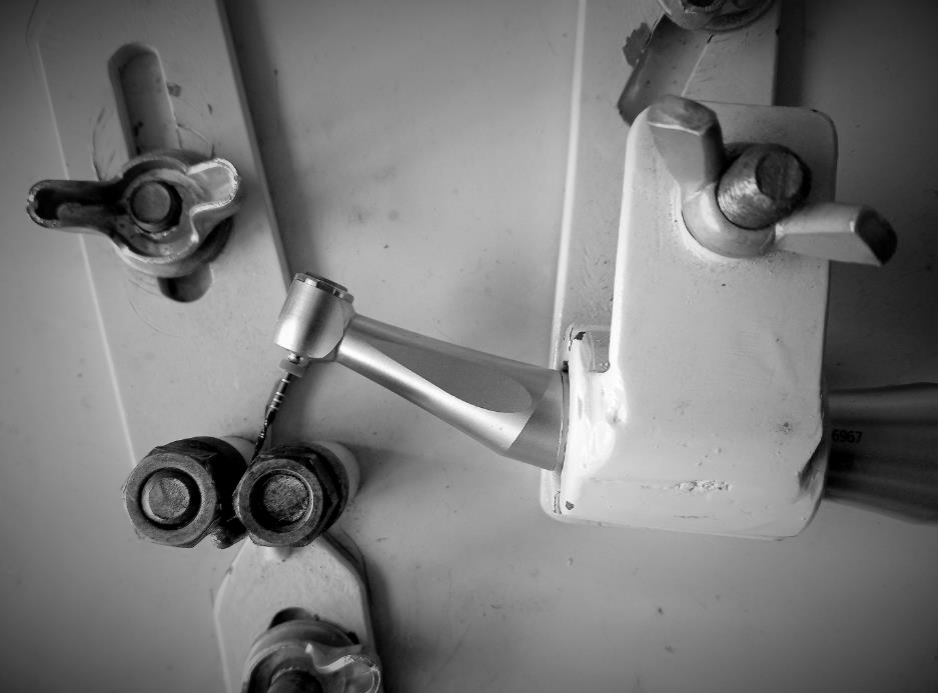
Cyclic fatigue resistance test

All instruments (n=18) were submitted to the cyclic fatigue resistance test. A steel device simulating a root canal with a bending angle of 60º and a radius of curvature of 5mm was used. All instruments were tested using a VDW Silver endodontic engine (VDW Dental), using the wave one all system until the instrument fractured (Figure 2). To minimize friction between the instrument and the artificial channel, a synthetic oil (SINGER, Cotia, Brazil) was used as a form of lubrication. For measurement, a digital stopwatch was used, which was stopped immediately when the fracture was verified. In order to increase the accuracy of the test, videos were taken during the test to confirm the data obtained. The number of cycles to fracture was calculated with the following formula: the number of cycles to fracture = rotations per minute (rpm) x time to fracture in seconds/60. To designate the rpm of the reciprocating movement wave one all, a record of 350 rpm was used, as mentioned in previous studies ^19^.

### Statistical Analysis

The data were initially analyzed using the Shapiro-Wilk test. Kruskal-Wallis test was performed for data analysis using SPSS 22.0 software (IBM, Armonk, New York, EUA). The level of statistical significance was considered p < 0.05.

## Results

The group I, which used a #15 Kerr File as the glide path, demonstrated the highest average time in seconds to fracture (308±97.75), which resulted in an average of 1800.55±570.23 cycles to fatigue. Group II used a specific reciprocating instrument for glide path (Wave One Glider), and obtained an average time in seconds for fracture of 274.33±127.11, which resulted in an average of 1600.27±741.48 cycles to fatigue. Group III, which did not use a specific instrument for glide path, presented the lowest data, with an average time in seconds for fracture of 253.33±78.56, generated in an average of 1477.77±458.28 cycles to fatigue. The data can be observed in detail in table 1.

The results showed no statistically significant difference between the tested groups. In addition, during the preparation of the teeth, the safety of the preparation protocols was observed, since there was no occurrence of instrument fracture in any of the groups.

**Table 1:** Results of the time and numbers of cycles required for the occurrence of fracture of the reciprocating instrument used on the chemicomechanical preparation

Groups	Median	Standard deviation	P value
Time (seconds)			
1	308,66	97,75	0,630
2	274,33	127,11	
3	253,33	78,56	
Cycles (fatigue cycle)			
1	1800,55	570,23	0,630
2	1600,27	741,48	
3	1477,77	458,28	

## Discussion

Numerous factors are associated with the resistance to cyclic fatigue of endodontic instruments. The aim of the study was to present two variables and their relationship in clinical practice: the use of different glide path instruments, and the reuse of reciprocating preparation instruments.

The term glide path has been used to designate a stage of endodontic treatment that consists in using instruments to keep the path of the root canal completely unobstructed. The cyclic fatigue resistance of reciprocating instruments for preparation with and without the use of the glide path instrument is a subject that has been addressed recently. A study aimed to analyze the association of the cyclic fatigue resistance of the reciprocating instrument used for root canal preparation. The results showed that creating a glide path did not affect the cyclic fatigue resistance of the tested reciprocating instruments ^20^. Following the same line of reasoning, and presenting similar results, another study aimed to compare the cyclic fatigue resistance of preparing reciprocating instruments with and without the use of two glide path instruments, the Pro Glider and Path File. The results showed that the creation of the glide path had no relationship with the cyclic fatigue resistance of the preparation reciprocating instruments ^16^.

To date, there is no previous study that has used a glide path reciprocating instrument to verify the cyclic fatigue resistance of the reciprocating preparation instrument, therefore, this work presents unpublished data, and the results of the present study cannot be directly compared. However, the present results demonstrate that the creation of a glide path, either with manual stainless steel instruments (K file #15) or with reciprocating instruments, is not related to the cyclic fatigue resistance of the reciprocating instruments used for preparation. These results have similarities with the results found in previous studies ^16^ ^,^ ^20^.

Some studies indicate that the use of instruments with reciprocating kinematics and heat treatment on the alloy can easily reach the working length during clinical activity, even without the use of the instrument to perform the glide path ^21^. According to the most contemporary results, there is no influence of the glide path on the cyclic fatigue resistance of reciprocating instruments for several reasons, such as the kinematics, since instruments with reciprocating kinematics have greater resistance to cyclic fatigue, also present higher torsional resistance ^22^, the evolution in the development of metallic alloys and the advent of heat-treated instruments, increasing flexibility, and reducing the stress generated in the instrument ^20^.

The other variable studied in this study was the reuse of reciprocating instruments for root canal preparation. The recommendation of the manufacturer of endodontic instruments is to use them once only. Studies have evaluated the influence of clinical use on the occurrence of deformation and fractures in final preparation instruments. The results showed that the resistance to cyclic fatigue decreases with clinical reuse for all instruments tested ^23^.

Other authors evaluated the cyclic fatigue of instruments after use in molars, and their results showed that clinical use reduced the resistance to cyclic fatigue of instruments when compared to new instruments ^24^. However, depending on the social and economic conditions in which the professional is inserted, the reuse of instruments is commonplace. An important survey studied the clinical conditions of endodontists who use automated systems for root canal preparation in the United States. The results indicated that 74% of the professionals who participated in the survey reuse the instruments, and 40.5% of the endodontists who work in private practice discard the instrument only after the third use ^6^. Comparing with the situation in another country, with different socioeconomic conditions from the previous study, the survey evaluated the reuse of endodontic instruments in South Africa. The results showed that 81.5% of participants reused automated NiTi endodontic instruments ^7^.

In addition to the data already presented, a systematic review aimed to evaluate the incidence of fracture of automated endodontic instruments. The study indicated a low rate of instrument fracture and cites as one of the characteristics of this result the continuing education of professionals, and the familiarization with the systems and work systematics. Moreover, this study suggests that further research should be conducted to develop protocols for reuse, in order to optimize the clinical, economic, and social significance ^25^.

The limitation of our study is the lack of standardization regarding the apical diameter of the selected dental elements. This fact is due to the clinical act to determine the apical diameter, which involves firstly the preparation of the middle cervical third, and the use of a stainless steel instrument that is as tight as possible in the working length. Such clinical conduct would impair the analysis of the cyclic fatigue resistance of the instruments, which was the objective of the study. As a suggestion, we point to the determination of the apical diameter by non-destructive means, such as the use of Micro-CT examination, which would enable an even more homogeneous sample. In addition, several studies with a similar theme ^5^ ^,^ ^13^ ^,^ ^16^ ^,^ ^23^ ^,^ ^24^ presented a *n* with approximately 10 to 12 teeth/instruments per group in their sample size calculations. Our study, based on parameters and a sample size calculation, obtained an estimate of 6 instruments per group. As a suggestion, we point to the need to carry out more studies with larger sample size, in order to reduce possible biases and to obtain more robust scientific evidence with greater statistical power.

In addition, due to the purpose of analyzing the reuse of automated instruments with or without the use of glide path instruments, the selected dental elements were lower molars with little curvature in their roots. The data found in this study cannot be extrapolated to teeth with large degrees of curvature, or those present anatomical complexities that make root preparation more challenging. Based on this, we suggest that another studies, with similar methodology, be carried out in order to analyze the points raised (instrument reuse and the use of glide path) in other dental groups, and also in dental elements with more challenging anatomies.

Our study corroborates a previous work that used reciprocating instruments in three posterior dental elements with safety and low incidence of fracture. Moreover, it contributes to the construction of protocols for the reuse of instruments in a safe manner, so that the clinician can be guided in clinical practice, according to the social and economic reality in which the professional is inserted. Clinical studies should be conducted to study the reuse and the creation of safe and reproducible clinical protocols. Moreover, our study presents important glide path situations, especially the use of reciprocating kinematic instruments for this purpose, which is consistent with other instruments used for this purpose, whether manual or rotary.

Within the limitation of an in vitro study, it was concluded that creating a glide path with a reciprocating instrument did not affect the cyclic fatigue resistance of the final preparation reciprocating instrument when compared to manual and rotary instruments. The reciprocating instrument was used for final preparation and subsequently safely reused on three posterior teeth. No fractures occurred in any of the groups tested.
